# Scaling of temporal correlations in densely connected networks of LIF neurons

**DOI:** 10.1186/1471-2202-12-S1-P249

**Published:** 2011-07-18

**Authors:** Jesús Manrique, Alfonso Renart, Jaime de la Rocha, Néstor Parga

**Affiliations:** 1 Dpto. Física Teórica, Universidad Autónoma de Madrid, 28049,Madrid, Spain; 2 Champalimaud Centre for the Unknown, 1400-038 Lisbon, Portugal; 3 Institut d'Investigacions Biomèdiques August Pi i Sunyer(IDIBAPS), 08036, Barcelona, Spain

## 

**Figure 1:**
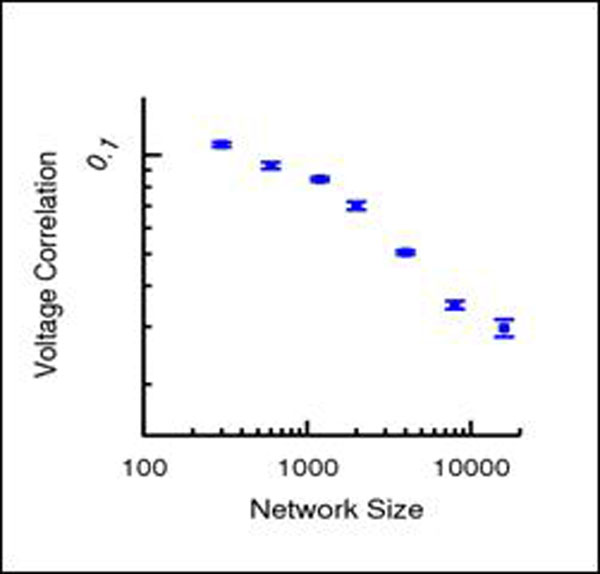
Average correlation coefficient of the membrane potentials for EE pairs.

It has been experimentally reported that neurons in cerebral cortex can fire in a decorrelated fashion despite presumably sharing a non-negligible fraction of their inputs[1,2]. Performing a scaling analysis where the synaptic strengths decrease as 1/sqrt(N), being N the number of neurons in the network, it has been analytically shown [1]that randomly connected networks of binary excitatory (E) ad inhibitory (I) neurons show average correlation coefficient of the activity which decreases as 1/N while correlations of the synaptic current components remain O(1). The above applies to densely connected networks in which the connection-probability does not depend on N[1], in contrast to sparse networks where it is the number of connections of a neuron which is kept constant[3]. Here we present a numerical study of the dynamics of random and densely connected EI networks of current-based leaky-integrate-and-fire neurons. Preliminary results show the existence of two regimes depending on the magnitude of the external drive onto the network: one in which average correlations do not systematically decrease with N (regime obtained with large external currents) and one in which average correlation of voltages (Fig. 1) and spike counts decrease with N (regime obtained with small external current). Preliminary data indicates that the latter regime occurs in the presence of strong correlations of the current components. The lag between excitatory and inhibitory spikes also decays with the network size. These results suggest that a mechanism of active decorrelation of synaptic inputs similar to the one previously found for binary networks, also exists in networks of integrate-and-fire neurons.
